# Impact of selective serotonin reuptake inhibitors on major salivary glands and mandibular alveolar bone; a histological, histochemical and biochemical study

**DOI:** 10.1186/s12903-026-07746-4

**Published:** 2026-02-12

**Authors:** Nehad M. Abd-elmonsif, Sherif Gamal, Bassant M. Bahgat

**Affiliations:** 1 Department of Oral Biology, Faculty of Oral and Dental Medicine, Future University in Egypt, Cairo, Egypt; 2 Research Labs Supervisor, Faculty of pharmacy, Future University in Egypt, Cairo, Egypt; 3 Department of Oral Pathology, Faculty of Oral & Dental Medicine, Egyptian Russian University, Cairo, Egypt

**Keywords:** Cipralex, Selective serotonin reuptake inhibitors, Major salivary gland, Alveolar bone, Interleukin 1 beta, Tumor necrosis factor alpha

## Abstract

**Background:**

Selective serotonin reuptake inhibitors (SSRIs) are a novel type of antidepressant (AD) that can be used as a first-line treatment for depression and other mental illnesses. Long-term AD use is becoming more frequent, with many people continuing to get therapy for extended periods of time. This surge has been linked to a number of health concerns, including an increased risk of fractures, lower bone mineral density in young adults, and a significantly elevated risk of dry mouth, all of which can have a negative impact on dental health and general quality of life. This study aimed to evaluate the effect of Cipralex (SSRI) on the histological structure of major salivary glands and alveolar bone, as well as its impact on pro-inflammatory cytokines interleukin 1 beta (IL-1β) and Tumor necrosis factor alpha (TNF-α).

**Methods:**

18 rats were randomly separated into two groups (*n* = 9 each): a control group and a Cipralex-treated group receiving 10 mg/kg/day orally by gavage for four weeks. Major salivary glands and mandibular molar region specimens were collected for histological evaluation using hematoxylin and eosin( H&E) staining. Alveolar bone specimens were stained with Masson’s trichrome (MT), and histomorphometric analysis of newly formed collagen area percentage was performed using ImageJ software. Serum IL-1β and TNF-α levels were quantified using enzyme-linked immunosorbent assay (ELISA).

**Results:**

The Cipralex group’s histological analysis showed that the architecture of the normal major SGs had deteriorated notably, and there were indications of bone resorption in the alveolar bone. There was a statistically significant decrease in the amount of newly produced collagen compared with controls (*p* < .05). Biochemical analysis showed a significant decrease in serum IL-1β levels in the Cipralex group (245.22 ± 5.04) versus control (262.67 ± 4.88), while TNF-α levels did not differ significantly between groups ( control ; 693.88 ± 8.65, cipralex; 695.84 ± 7.06) *p* = .607 .

**Conclusions:**

Long-term use of Cipralex may compromise the integrity of the salivary glands and raise the possibility of alveolar bone resorption. These results highlight the significance of dental and periodontal monitoring in patients undergoing long-term antidepressant treatment and emphasize possible oral health problems linked to long-term SSRI therapy.

**Graphical abstract:**

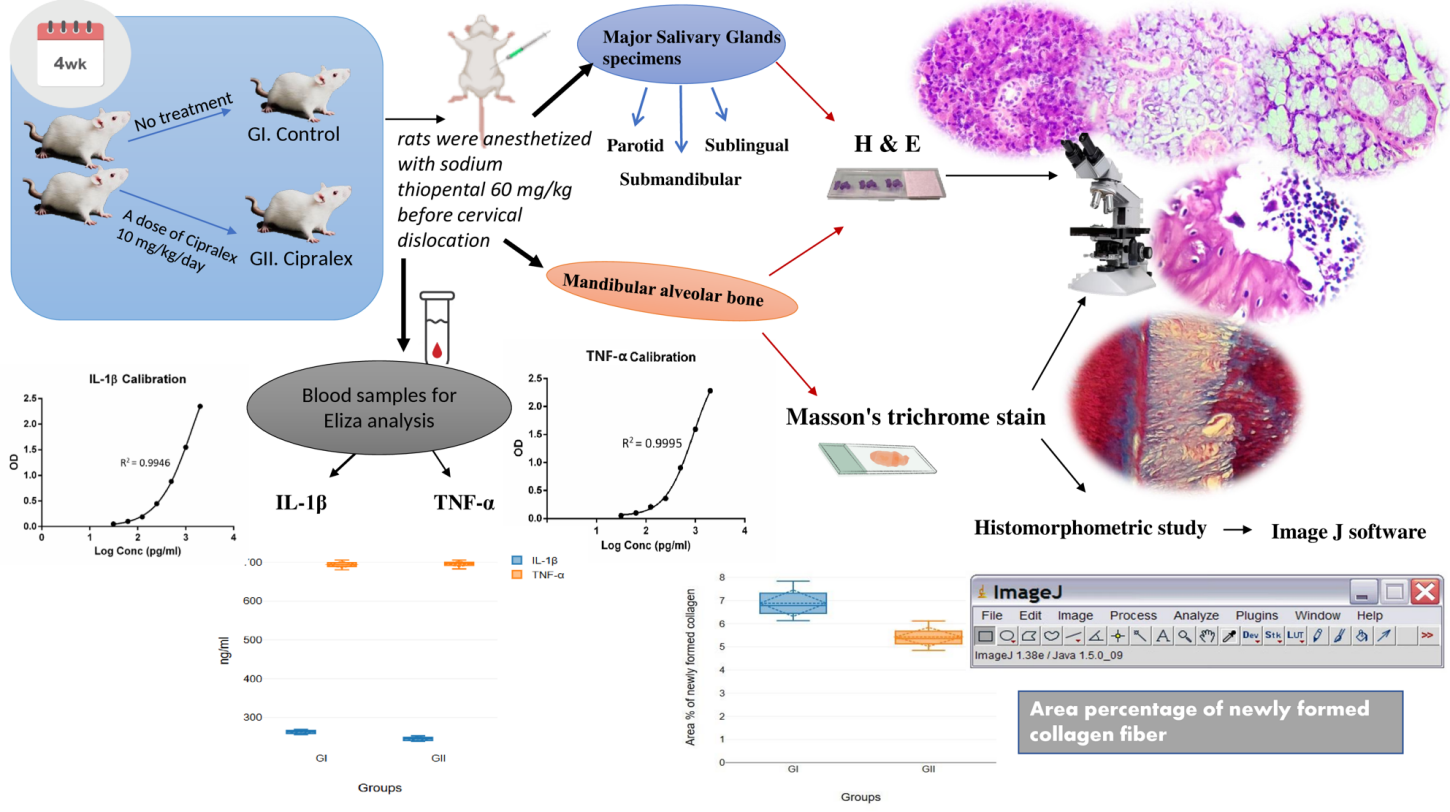

## Background

Depression is a common mental illness characterized by despairing mood, anhedonia, sleep problems or appetite, inactivity, and impaired attention. Approximately 5% of adults worldwide experience symptoms of depression. The World Health Organization estimates that depression affects about 280 million individuals globally and relates to an increase in suicide [1].

Depression can be caused by a variety of reasons, including stressful life events [2], borderline personality disorders [3], chronic sedative and hypnotic use, and deleterious effects from long-term use of β-blockers, antipsychotic medications, and isotretinoin [4, 5]. Moreover, psychological illnesses such as bipolar disorder, major depressive episodes, and seasonal affective disorders raise the vulnerability of depression [6]. Non-psychiatric illnesses such as hypothyroidism [7], Cushing disease [8], Parkinson disease [9], and multiple sclerosis [10] all increase the frequency and incidence of depression.

Antidepressants (ADs) serve an important role in treating depression. Selective serotonin reuptake inhibitors (SSRIs) are a new AD that has suit as first-line treatment for depression and several mental disorders because of its effectiveness and safety [11]. In the United States (US), 62% of ADs are used as SSRIs, which include escitalopram, citalopram, sertraline, and fluoxetine [12]. Concern over SSRI safety has been developing recently. An SSRI called escitalopram, which is marketed under the Cipralex brand, is used to treat anxiety and depression by reestablishing serotonergic function [13].

In the US, the third most prescribed medicine class is antidepressants, which includes selective serotonin reuptake inhibitors (SSRIs). Antidepressant use has increased by more than 400% since 1994, with an estimated 10% of Americans currently using them. Furthermore, the prevalence of long-term usage is rising; 14% of SSRI users have been on medication for more than ten years, and 60% of users have been on treatment for more than two years [14]. Multiple illness, such as a higher risk of fracture and reduced bone mineral density in adolescents and young adults, are increasingly being identified as a result of the sharp rise in AD use. Additionally, SSRI usage is linked to a higher incidence of fracture in at-risk groups (such as post-menopausal women) [15–18], surpassing other medications that are not recommended, such as proton pump inhibitors [19] and steroid hormones [20].

Even the safest ADs might have negative side effects with prolonged use. One of the biggest problems in clinical practice is drug-induced organ damage [21]. Studies undertaken during clinical trials frequently do not accurately reflect the true outcomes produced by medications in actual clinical practice since they are generally brief in duration and do not represent all types of people with real-life diseases [22]. Newer ADs that have less side effects than traditional ones, including monoamine oxidase inhibitors or tricyclic antidepressants (TCAs), have been developed in recent years [23]. Since not all ADs have antioxidant activity, etiologic variables like oxidative stress have been implicated in the incomplete understanding of ADs effects [24]. Their usage has been linked to abnormalities in aerophilic stress indicators, mononuclear cells, and red blood cells (RBCs) in postmortem brains, urine, and cerebrospinal fluid (CSF) [25–27]. Long-term use of ADs, particularly SSRIs, has been linked to an increased risk of type 2 diabetes [28]. In addition, most antidepressants are associated with a significantly increased risk of dry mouth [29].

According to recent studies, escitalopram (Cipralex) can affect peripheral tissues and modify molecular pathways linked to oxidative stress, inflammation, and neuroendocrine signaling in addition to its classical action as an SSRI. Chronic escitalopram has been demonstrated in animal experiments to modify tryptophan metabolism, suppress systemic inflammatory markers, and alter the composition of the gut microbiota, suggesting wider immunomodulatory and metabolic effects [30]. Furthermore, escitalopram has been shown to modify intracellular signaling pathways linked to neuroplasticity and stress resilience as well as control neuroendocrine peptides in peripheral tissues [31].

Saliva is produced, modified, and released into the mouth cavity by the salivary glands (SGs), which are exocrine glands. They are separated into essentially two classifications: the minor salivary glands, which line the mucosa of majority of the oral cavity, and the major salivary glands, which are composed of the parotid, submandibular (SMG), and sublingual glands (SLG) [32].

a systematic review shows that SSRIs contribute to oral manifestations like xerostomia (dry mouth), taste dysfunction, and deteriorated jawbone quality, highlighting effects on oral tissues that may in turn involve salivary gland function, despite a shortage of direct experimental evidence on SSRIs and salivary gland biology [33].

Cipralex’s impact on periodontal conditions and alveolar bone levels in depressed individuals was carefully assessed in a recent clinical case-control research. Remarkably, compared to non-users, Cipralex use was linked to better periodontal clinical signs and decreased marginal bone loss [34].

Cytokines are signaling molecules that facilitate cell-to-cell contact and are essential for inflammation and immunological responses. In addition to being vital for host defense, interleukin 1β (IL-1β) is a major modulator of the inflammatory response and contributes to tissue damage in both acute traumas and chronic illnesses. Tumor necrosis factor alpha (TNF-α) is another significant pro-inflammatory cytokine that affects the release of other cytokines, such as interleukin 6 (IL-6), and is involved in immunological responses [35]– [36].

Emerging research suggests that SSRIs may influence inflammatory processes in anxious and depressed individuals. Clinical trials have found changes in peripheral inflammatory markers following Cipralex and other SSRI medication, including reductions in numerous pro-inflammatory cytokines, indicating that these drugs may have an immunomodulatory role [37].

A meta-analysis of clinical studies found that SSRI treatment significantly lowered levels of pro-inflammatory cytokines, such as IL-1β and TNF-α, in patients with severe depressive disorder. These data lend support to the theory that, in addition to antidepressant activity, SSRIs may have anti-inflammatory and immunomodulatory properties [38].

A recent in vitro investigation found that escitalopram boosted the production of TNF-α and IL-6 in macrophages, indicating that this SSRI can regulate cytokine expression in certain immune cell types [39].

To far, few animal studies have simultaneously investigated the effects of SSRIs on major salivary glands and mandibular alveolar bone, particularly in relation to inflammatory and oxidative stress pathways. Given the prevalence and duration of use of these drugs, this is a critical gap in the literature.

Thus, the current study aimed to determine how cipralex affected the histological structure of the mandibular alveolar bone and major salivary glands, as well as to measure alterations in the expression of inflammatory cytokines (IL-1β and TNF-α) in an animal model.

We hypothesized that chronic cipralex use would trigger inflammatory alterations in salivary gland tissues and adversely impact alveolar bone integrity via cytokine-mediated mechanisms.

## Materials and methods

### Study settings

The present investigation included 18 adult male albino rats weighed between 170 and 180 g. The animals were obtained from the animal house of Faculty of Pharmacy, Future University of Egypt, Cairo, Egypt. Rodents were staying in clean, well-ventilated cages, fed a standard laboratory diet, and subjected to a cycle of 12-hours of light and darkness.

Healthy adult male rats within the designated weight range, free of drug or experimental procedure exposure, and displaying typical eating and activity patterns were the requirements for inclusion. Female rats, rats exhibiting symptoms of disease, congenital defects, injuries, or aberrant behavior, as well as rats that fell outside of the specified weight range, were all excluded.

### Sample size

The sample size was determined using MedCalc^®^ statistical software version 12.3.0.0 (MedCalc^®^ software, Ostend, Belgium). According to earlier studies [40, 41], the effect size was 0.79. statistical calculation using a 95% confidence interval, an 80% study power, and a 5% α error. For all groups, the predicted sample size (n) was 18. Each group’s mortality % was calculated and documented (no losses occurred).

### Ethical approval

Albino rats received housing and engaged in for experiments following The ARRIVE Guidelines for Reporting Animal Research, in accordance with the Research Ethics Committee at the Faculty of dentistry, Future University (REC-FODM) under number FUE.REC (42)/6-2025.

### Pharmaceutical drug

Tablets of Cipralex (formerly known as Escitalopram) (H-Lundbeck A/S, Valby, Denmark) were purchased at an on-site pharmacy in Egypt. Escitalopram (10 mg) is present in each tablet. For four weeks, the experimental dose, which is 10 mg/kg/day diluted in 0.1 ml/100 g of 0.9% saline solution, was administered orally by oral gavage [42].

### Study design

The 18 rats were randomly divided into two groups: Group I (Control) (no treatment) and Group II (Cipralex) (received a dose of Cipralex 10 mg/kg/day, diluted in 0.1 ml/100 g 0.9% saline solution, administered orally by gavage for 4 weeks) [42]. To guarantee unbiased group distribution, randomization was carried out utilizing a computer-generated random sequence. To reduce observational bias, researchers observing the microscopic and histological investigations were blind to the group assignments.

Before cervical dislocation, rats were euthanized with sodium thiopental (STP, 60 mg/kg (EIPICO, Egypt) intraperitoneal injection) and starved overnight at the final day of the 4-week trial. Specimens of the mandibular molar area and major SGs (Parotid SG, SMG and SLG) were prepared for histological analysis.

### Preparation of tissues for histological evaluation

#### Preparation of major SGs specimens

SGs tissues were stored in a 10% neutral buffered formalin solution for 48 h. After being washed in three xylene changes for an hour each at room temperature and dehydrated in progressively higher ethanol grades, the samples were embedded in paraffin wax. After being cleaned and sterilized 3 serial slices with thickness 4–5 μm were selected and placed on glass slides.

#### Preparation of mandibular alveolar bone

The specimens were dehydrated and embedded in paraffin after being immersed in 10% ethylene diamine tetra-acetic acid (EDTA) for four weeks to decalcify them. The specimen was gently probed with a needle to gauge its softness and flexibility in order to determine the decalcification endpoint.

The decalcification process is considered effective when the tissue is soft and easily pierced with no resistance. This was carried out by an expert technician. Hematoxylin and Eosin (H & E) was used for histopathological analysis on slices that were 4–5µ thick. Masson’s trichrome (MT) stain was used to identify immature (newly created) collagen (blue color) and mature (old) collagen (red color).

### Histomorphometric study

Sections stained with MT were used to calculate the area % of freshly formed immature collagen.

Each group had a total of nine specimens examined. Three non-overlapping fields were examined at ×200 magnification in each representative segment that was chosen from each specimen.

ImageJ v. 1.43u (National Institutes of Health, Bethesda, MD, USA) was utilized for image analysis. Based on the distinctive blue staining of Masson’s trichrome, the Color Threshold tool was used to establish the threshold for detecting newly formed immature collagen. To maintain uniformity and reduce observer bias, threshold values were maintained for each image.

The studied regions were consistently chosen from the mandibular molar area, specifically the oblique and periapical portions of the mandibular molars, alongside avoiding locations with artifacts or sectioning defects. In order to ensure standardization, both the experimental and control groups utilized the same anatomical landmarks.

### Blood sample collection

A heart puncture was used to get blood at the completion of the experiment. To ensure adequate coagulation, blood samples were allowed to sit at room temperature for 30 min. After centrifuging blood samples for 10 min at 2000 rpm, the serum was extracted and gathered. For future investigations, serum samples were kept at -20 °C.

### Biochemical analysis

Different biomarkers’ serum levels were determined using the Enzyme-Linked Immunosorbent Assay (ELISA). Levels of interleukin-1β (IL-1β) were identified using Rat IL-1β ELISA kits (Elabscience, USA. CAT No. E-EL-R0012, Detection Range; 31.25–2000 pg/mL) in compliance with the guidelines provided by the manufacturer.

Levels of TNF-α were identified using Rat TNF-α ELISA kits (Elabscience, USA. CAT No. E-EL-R2856, Detection Range; 15.63–1000 pg/mL) in compliance with the guidelines provided by the manufacturer.

Proinflammatory cytokine concentrations were examined and measured using optical density (OD). The absorbance was measured at 450 nm wavelength. ELISA assays were carried out at the Nawah Scientific Center in Cairo, Egypt.

Each experimental and control group contained nine rats, with each animal representing one biological replication. Serum samples from each rat were collected and examined independently.

Each serum sample was measured in technical duplicate for the ELISA tests, as specified by the manufacturer. The final value for each animal was derived using the mean of the two technical replicates. Biochemical results are expressed as the mean ± SD of nine biological replicates per group.

### Statistical analysis

The area % of newly generated collagen and biochemical data were statistically assessed using SPSS version 17 (IBM Corporation, Somers, New York, USA). The distribution of data was evaluated using the Shapiro-Wilk normality test. All data were normally distributed, and a parametric T-test was used. The results were expressed as mean ± SD, and p-values less than 0.05 were considered significant.

## Results

### Histological results

#### H&E results of major SGs

##### Parotid G findings

**Fig. 1:**
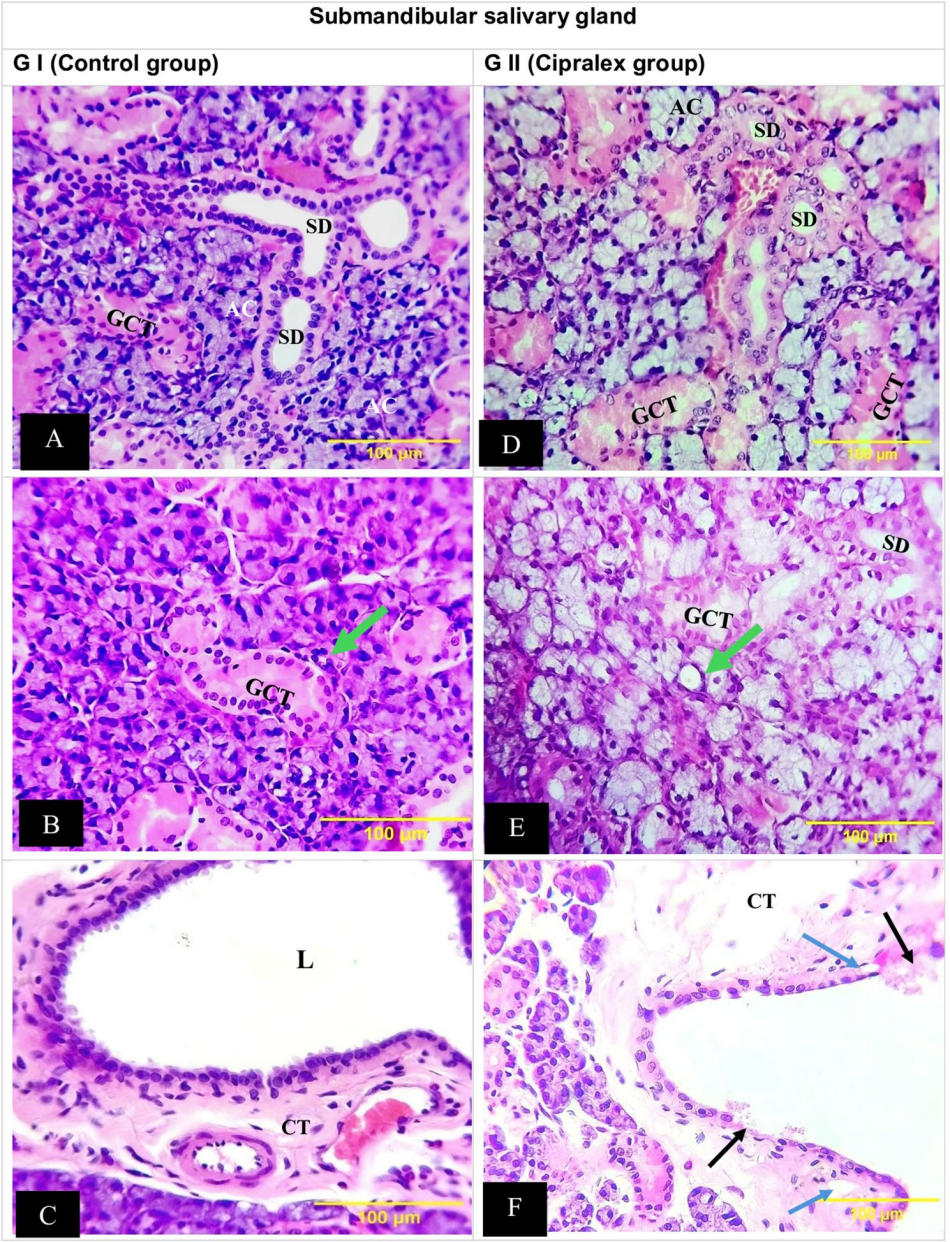
Photomicrographs of parotid gland: (**A **&** B**) Control Group; densely packed serous acini, with uniformly pigmented cytoplasm free of vacuolations and with spherical nuclei (AC), normal intercalated duct (black arrow), striated duct (SD). (**C**)Excretory duct of control group; lining of pseudostratified columnar epithelium (arrow), surrounding wide lumen (L). (**D**)Cipralex group; serous acini lost their integrity and form, showed uniform basophilic staining (AC), normal histology of intercalated duct (black arrow), striated duct (SD).(**E**)Cipralex group; wide adipose regions in the gland structure occasionally showed signs of acini degeneration (AD). (**F**)Excretory duct of Cipralex group; atrophied and deformed epithelium lining (arrow) with sporadic secretion that was stagnant in the lumen (L). (H&E, Orig. Mag.200x)

Examining the GI (control group) samples showed that the parotid G’s histological characteristics were normal, with uniformly pigmented cytoplasm free of vacuolations and densely packed serous acini with spherical nuclei. There were ducts of different sizes with different epithelial linings, including intercalated (ID), striated (SD), and excretory (ED) ducts (Fig. 1A, B & C).

There were obvious indications of degenerative alterations in GII (Cipralex group). Few vacuoles were visible, and the serous acini lost their integrity and form. Normal histology was shown by ID and SD, and the nuclei of serous acini showed uniform basophilic staining (Fig. 1D). Wide adipose regions in the gland structure occasionally showed signs of acini degeneration (Fig. 1E). Additionally, ED showed atrophied and deformed epithelium lining with sporadic secretion that was stagnant (Fig. 1F).

##### SMG findings

**Fig. 2:**
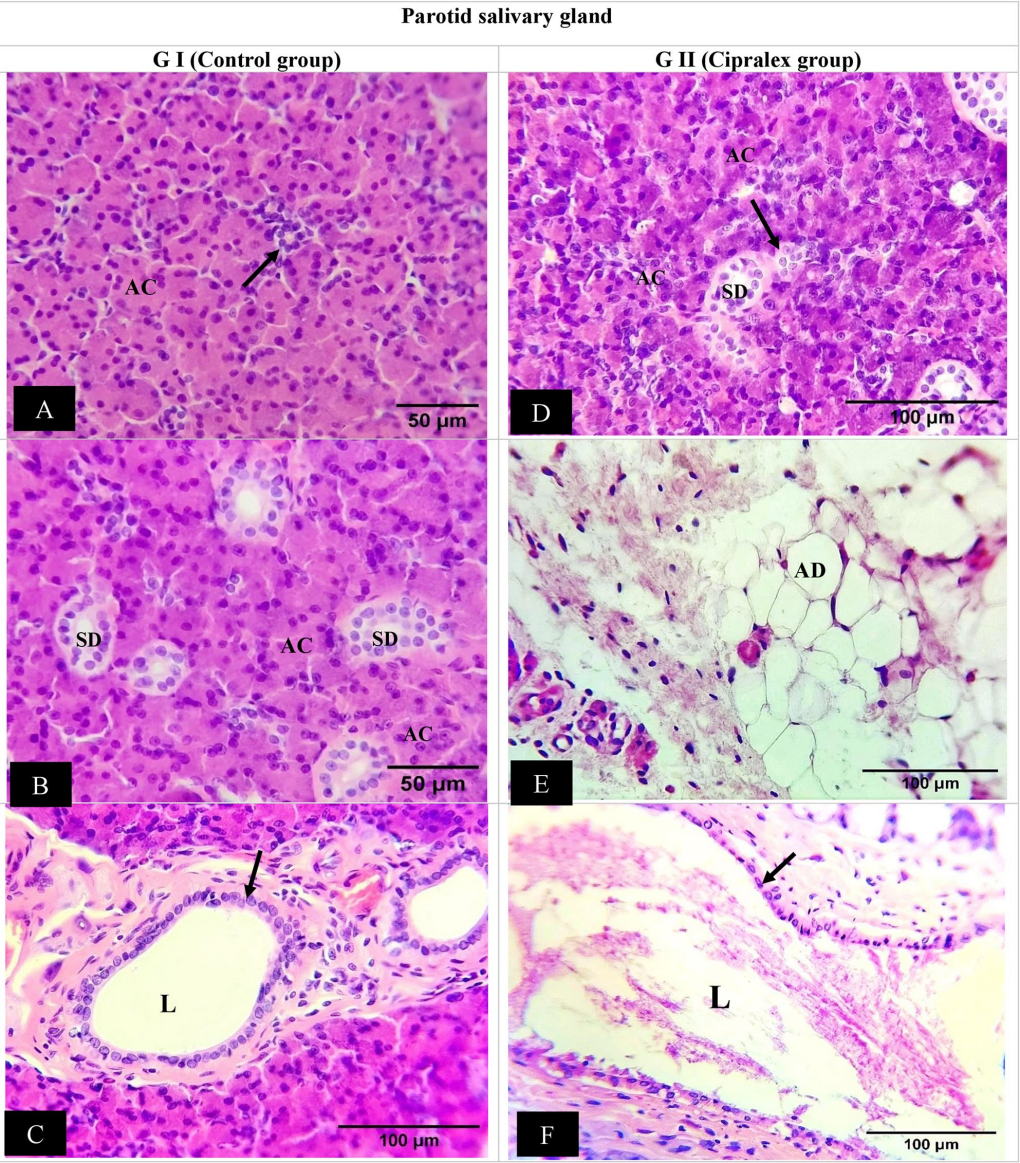
Photomicrographs showing submandibular salivary gland: (**A **&** B**) Control Group; seromucous acini are pyramidal in form, with nuclei positioned at the base (AC). Granular convoluted tubules situated between acini formed of columnar epithelium with many secretory granules in its cytoplasm (GCTs) and intercalated duct (green arrow), striated duct (SD). (**C**)Excretory duct of control group; pseudostratified columnar cells encircling a broad lumen (L), surrounded by normal connective tissues stroma (CT). (**D **&** E**)Cipralex group; seromucous acini with intracytoplasmic vacuolization. Intercalated duct revealed a decrease in cell height and a widening of the lumen (green arrow), striated ducts (SD) and Granular convoluted tubules (GCT) were partially degenerated and vacuolized. (**F**) Excretory duct of Cipralex group; vacuolization (blue arrows) as well as thinning and loss of integrity of the epithelial lining (black arrows). Significant deterioration was seen in the connective tissue stroma (CT). (H &E,Orig. Mag.200x)

GI (Control) histological sections revealed that the rat SMG was structurally normal. The cells of the seromucous acini are pyramidal in form, with nuclei positioned at the base. Granular convoluted tubules (GCTs) and ID, SD, and ED make up the duct system. Columnar cells with distinct basal striations and central nuclei lined the SD. In the rat SMG, the GCT was situated between acini and SDs. A straightforward columnar epithelium with many secretory granules in its cytoplasm made up the GCT wall (Fig. 2A & B). The ED’s epithelium is made up of pseudostratified columnar cells encircling a broad lumen. Additionally observed was normal connective tissue (CT) stroma (Fig. 2C).

Degeneration was seen in various areas of GII’s SMG. Intracytoplasmic vacuolization was observed in seromucous acini. ID revealed a decrease in cell height and a widening of the lumen. Additionally, SDs and GCTs were partially degenerated and vacuolized. Some of the eosinophilic granules in GCT disappeared (Fig. 2D&E). The EDs displayed vacuolization as well as thinning and loss of integrity of the epithelial lining. Significant deterioration was seen in the CT stroma (Fig. 2F).

##### Major SLG findings

**Fig. 3:**
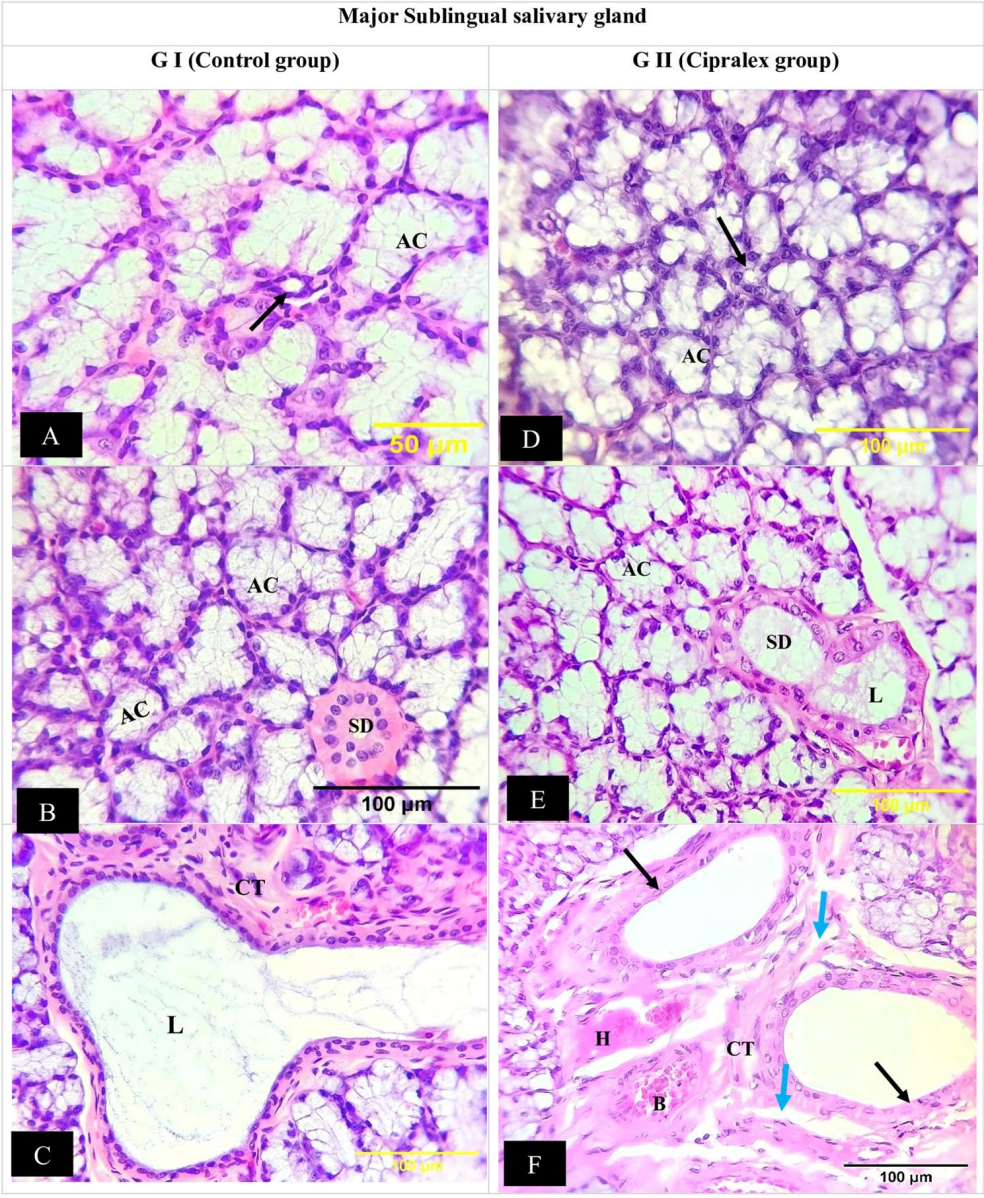
Photomicrographs of sublingual glands: (**A **&** B**) Control Group; normal mucous acini (AC), intercalated duct (black arrow), striated duct (SD). (**C**)Excretory duct of control group; wide lumen (L), normal connective tissue stroma (CT).(**D **&** C) **Cipralex group; mucous acini illustrated some vacuolation (AC), intercalated duct exhibited no discernible degenerative alterations (black arrow), striated duct lining cells appeared to have lost their basal striations, had cytoplasmic vacuolations, and were shorter in height (SD). Most duct lumens were dilated and filled with uniform, pink stagnant secretions (L).(**F**)Excretory duct of Cipralex group; loss of pseudo-stratification (black arrow). Connective tissue (CT) revealed regions of degeneration (blue arrows) and hyalinization (H), while nearby blood arteries seemed dilated and packed with RBCs (B). (H&E, Orig. Mag.200x)

The control group’s SLGs were made up of normal mucous acini. Mucous cells ranged in morphology from cuboidal to columnar, with oval nuclei that protruded toward the base of the cell. The light coloring of the mucous acini made them noticeable. They were elongated or tubular structures rather than spherical ones. ID, SD, and ED made up the duct system, which was surrounded by normal CT stroma (Fig. 3A, B, and C).

The mucous acini of SLG illustrated some vacuolation in the Cipralex group. IDs exhibited no discernible degenerative alterations and were like those of the control group (Fig. 3D). There were some changes to the SDs; the lining cells appeared to have lost their basal striations, had cytoplasmic vacuolations, and were shorter in height. Lumens of the ducts were dilated and contained uniform secretions, resulting in a broad, stagnant lumen (Fig. 3E).

In several places, the ED seemed to have lost their pseudo-stratification. CT revealed regions of degeneration and hyalinization, while nearby blood arteries showed dilatation and packed with RBCs (Fig. 3F).

#### Alveolar bone H& E results

**Fig. 4:**
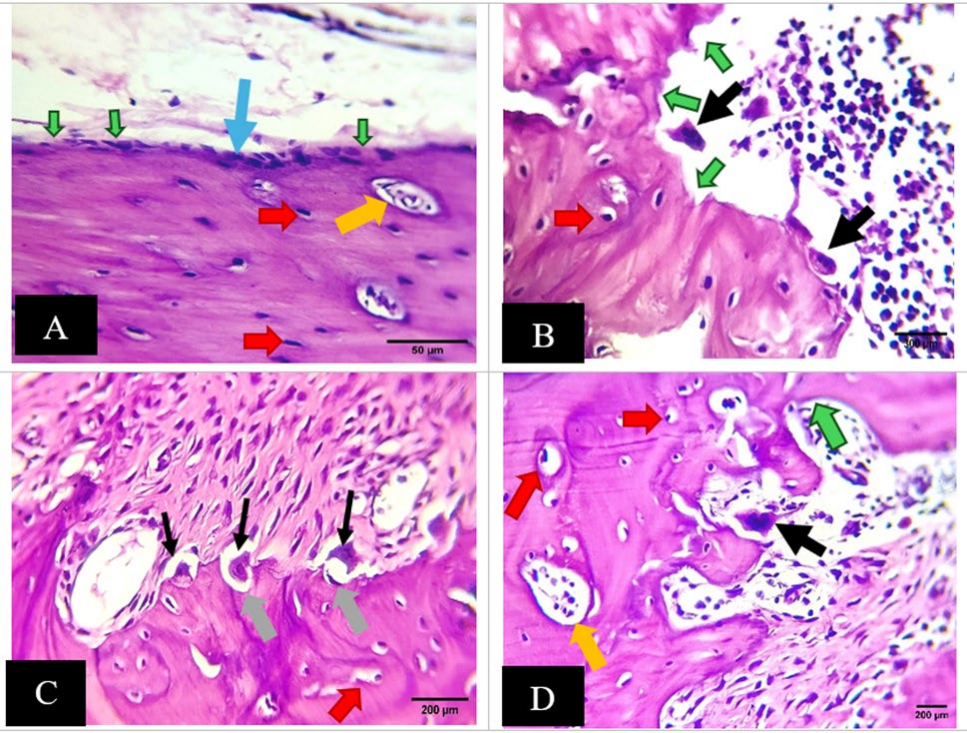
Photomicrographs of alveolar bone: (**A**)control group; a smooth alveolar border (green arrows), normal-sized marrow cavities (orange arrows), some osteoblasts looked flattened, while others were plump (blue arrow). osteocytes seemed normal (red arrows). (**B**,**C**&**D**)Cipralex group; The alveolar edge is uneven (green arrows), the marrow cavities appeared to be increased (orange arrow). A few osteocytes had empty lacunae, whereas others seemed to have expanded ones (red arrows). Numerous osteoclasts were found (black arrows) resting in a depression that resembled a bay (gray arrows). (H&E, Orig. Mag.400x)

The control group GI displayed normal-sized marrow cavities and a smooth alveolar border. Some osteoblasts looked flattened, while others were plump. There were no visible osteoclasts, and the osteocytes seemed normal (Fig. 4A).

The alveolar edge of the Cipralex group GII was uneven, and the marrow cavities appeared to be increased. A few osteocytes had empty lacunae, whereas others seemed to have expanded ones. Numerous osteoclasts were found resting in a depression that resembled a bay (Fig. 4B, C & D).

### Alveolar bone histochemical (MT) results

**Fig. 5:**
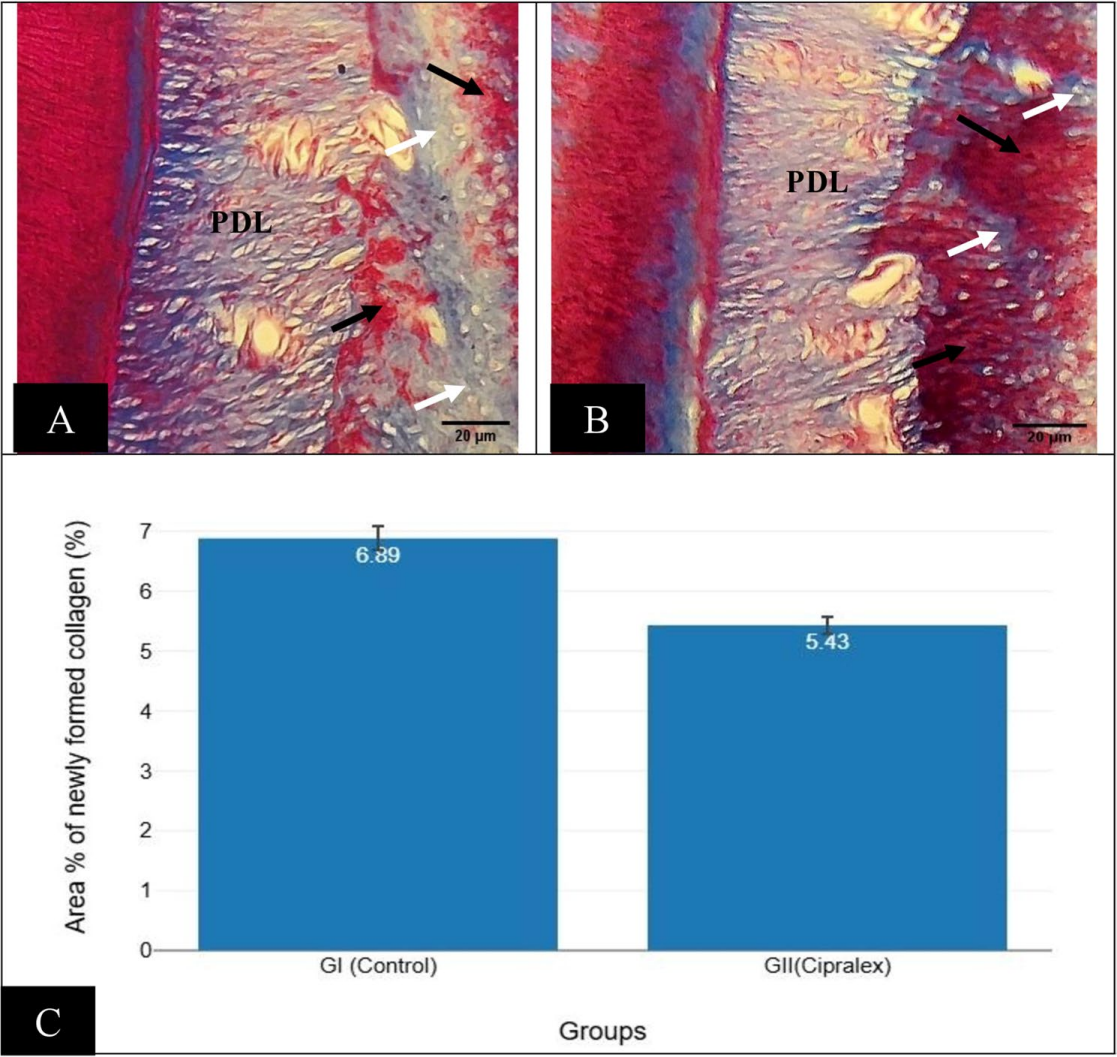
(**A**)Photomicrograph of control group showing alveolar bone and periodontal ligament (PDL) area; equal amounts of mature (red stained) (black arrows) and immature (blue stained) collagen fibers (white arrows).(**B**)Photomicrograph of Cipralex group showing alveolar bone and periodontal ligament (PDL) area; more mature bone (red stained) (black arrows) than immature bone (blue stained) (white arrows) (Masson trichrome, Orig. Mag.200x). (**C**) Barblot with error bar representing area percentage of newly formed collagen fibers regarding groups; control group (GI), Cipralex group (G II)

The control group had equal amounts of mature (red stained) and immature (blue stained) collagen fibers (Fig. 5A), but the Cipralex group had more mature bone (red stained) than immature bone (blue stained) (Fig. 5B). Healthy bone is indicated by newly generated collagen fibers that are blue stained.

### Area percentage of newly formed collagen

The difference between GI (6.89 ± 0.6) and GII (5.43 ± 0.43) Area % of newly formed collagen was statistically significant with *p* = < 0.001 with 95% confidence interval (Table 1; Fig. 5C).

**Table 1:** Showing the mean ± SD values, results of t-test of different studied groups regarding newly formed collagen area %, IL-1β and TNF-α

	Groups	*N*	Mean ± Std.	Cohen’s d effect size	t-test	confidence interval	*p*-value
Area % of newly formed collagen	GI (Control)	9	6.89 ± 0.6	2.80 (Very large )	5.89	95%	< 0.001**
	GII (Cipralex)	9	5.43 ± 0.43				
IL-1β	GI (Control)	9	262.67 ± 4.88	3.52 (Very large)	7.46	95%	< 0.001**
	GII (Cipralex)	9	245.22 ± 5.04				
TNF-α	GI (Control)	9	693.88 ± 8.65	-0.25 (Small)	-0.52	95%	*p* = .607
	GII (Cipralex)	9	695.84 ± 7.06				

** highly significant at *p*-value < 0.05

### The serum level of the pro-inflammatory biomarkers

**Fig. 6:**
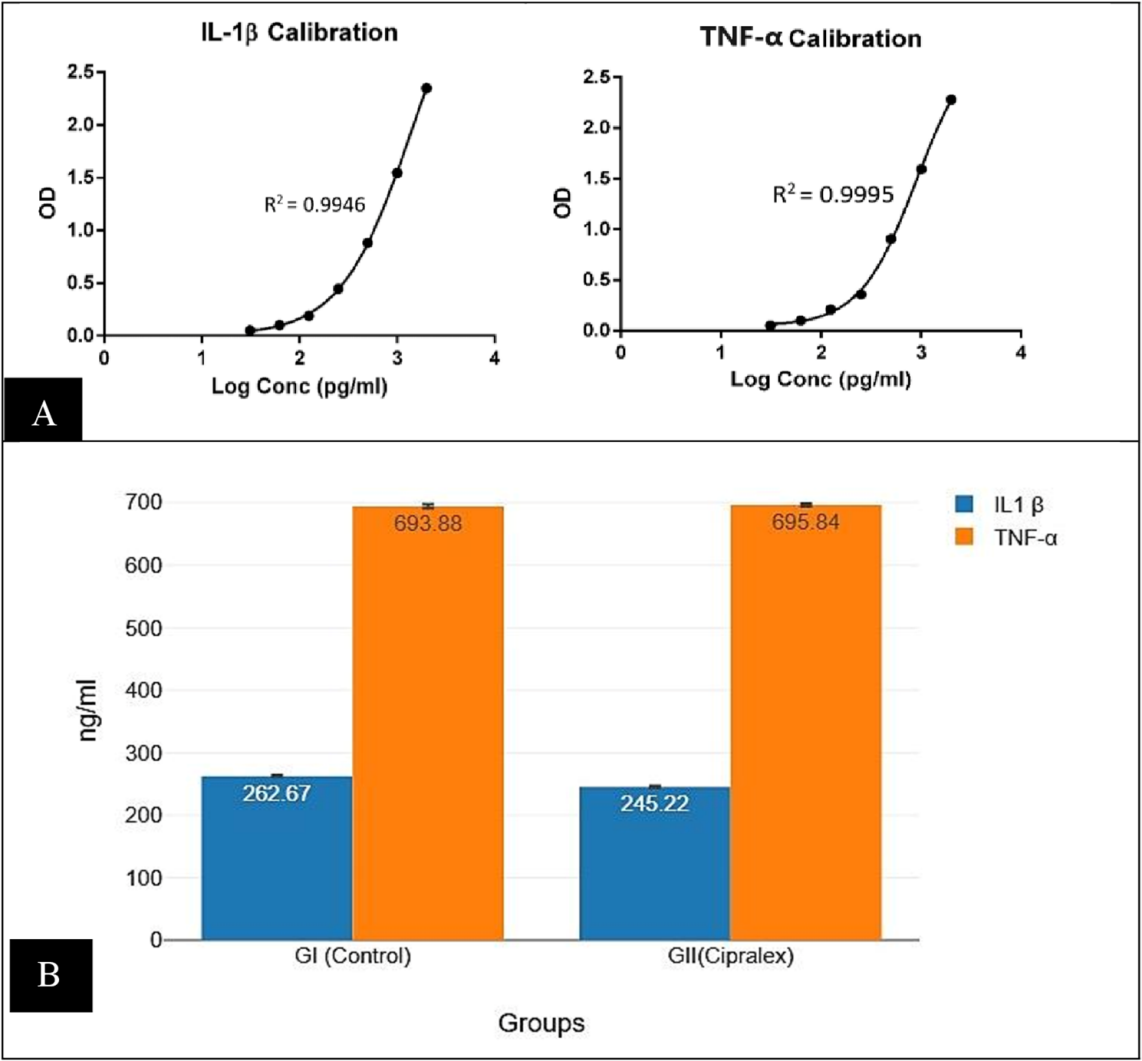
**(A)** Calibration curves of optical density (OD) measurements of Elisa for IL-1β and TNF-α. **(B)** Barblot with error bar representing serum levels of IL-1β and TNF-α regarding groups; control group (GI), Cipralex group (G II)

The Rat IL-1β and TNF-α solid-phase sandwich ELISA is intended to quantify the quantity of the target that is bound between a corresponding antibody. Calibration curves were created when the optical density (OD) was determined. (Fig. 6).

#### IL-1β

According to a two-tailed t-test for independent samples (assuming equal variances), there was a statistically significant difference between GI (262.67 ± 4.88)and GII (245.22 ± 5.04) in relation to the dependent variable IL-1β (*p* < .001) with 95% confidence interval (Table 1; Fig. 6).

#### TNF-α

With *p* = .607, a two-tailed t-test for independent samples revealed that there was no statistically significant difference between GI (693.88 ± 8.65) and GII (695.84 ± 7.06) regarding the dependent variable TNF-α with 95% confidence interval (Table 1; Fig. 6).

## Discussion

Since depression affects an extensive portion of the population, it is a serious public health concern [43]. According to a 2019 World Health Organization research, 23 million children and adolescents, out of 280 million people worldwide, suffer from depression [44]. The most often recommended ADs for this condition are SSRIs [45]. In this study, we assess how Cipralex, an SSRI, affects major SGs and alveolar bone, and we document how it affects serum levels of TNF-α and IL-1β. The results demonstrated that Cipralex has a detrimental effect on the tissues under study, lowering IL-1β levels while having no effect on TNF-α levels.

According to recent research, Cipralex causes degenerative alterations through oxidative stress, apoptosis, and mitochondrial dysfunction. Numerous experimental investigations have shown that SSRIs, such as escitalopram, might damage mitochondrial integrity, resulting in decreased ATP synthesis and elevated formation of reactive oxygen species (ROS). It has been demonstrated that these mitochondrial disruptions trigger intrinsic apoptotic pathways, which are characterized by the release of cytochrome c and the activation of caspase, ultimately leading to cellular degeneration in non-neuronal tissues [46, 47]. The architectural degradation seen in the major SGs in the current investigation is mechanistically supported by these findings.

Furthermore, oxidative stress has been found to be a crucial mediator of tissue damage triggered via antidepressants. Following long-term escitalopram administration, recent in vivo studies have shown elevated lipid peroxidation and decreased antioxidant enzyme activity, indicating a redox imbalance that exposes glandular tissues at risk for structural damage [48, 49]. The notable architectural disruption observed in the Cipralex-treated group may be explained by these oxidative changes, which may affect cellular membranes, acinar cell structure, and ductal integrity. These findings are in line with the deterioration of pancreatic tissue shown by Salama and Tayel [50], suggesting that escitalopram-induced toxicity may affect several exocrine glands.

On the other hand, escitalopram may have antioxidant or protective effects in certain experimental settings, especially in neural tissues or disease models with pre-existing oxidative damage, according to some recent research. In models of neuroinflammation and ischemia injury, for example, escitalopram has been demonstrated to reduce oxidative stress parameters and enhance mitochondrial function [51, 52]. This seeming conflict reveals how escitalopram’s biological actions are dose-dependent and tissue-specific. Thus, although escitalopram may have neuroprotective benefits in some situations, the current research suggests that long-term treatment can have detrimental structural effects on the tissues of the salivary glands.

Adult male rats were used as the preferred experimental model for this investigation to prevent any potential hormonal effects associated with the gender of the female rat [53].

According to Robbins et al. [54], cytoplasmic vacuolations in duct and acinar cells are a biological response to toxic compounds, wherein the materials build up in the vacuoles and are prevented from interfering with the metabolic function of the cell.

According to recent findings, cipralex and other SSRIs can cause intracellular stress reactions that are frequently linked to the creation of cytoplasmic vacuoles, such as endoplasmic reticulum stress, oxidative damage, and autophagy dysfunction. According to experimental data, long-term exposure to escitalopram (cipralex) raises ROS and damages organelle integrity in non-neuronal tissues, resulting in vacuolar degeneration and compromised cellular metabolism [47, 48]. These results are consistent with established strategies of drug-induced cellular damage, supporting the concept that vacuolation in the current study is a protective but pathological attempt by salivary gland cells to sequester hazardous compounds and suppress metabolic consequences.

According to recent research, SSRIs, such as escitalopram, may have a detrimental effect on bone health by stimulating bone resorption and hindering bone formation. Reduced bone mineral density, changed bone microarchitecture, and elevated markers of bone turnover have all been linked to long-term SSRI exposure, suggesting an imbalance in bone remodeling [55, 56]. Our histology observations of alveolar bone resorption in the Cipralex-treated group are consistent with these results. While central serotonin has different impacts on bone metabolism, SSRIs may mechanistically raise peripheral serotonin levels, which suppress osteoblast activity and promote osteoclast-mediated bone resorption [57].

Our study’s histological analysis of the Cipralex group’s alveolar bone revealed evidence of bone resorption, which is consistent with Wadhwa et al. [58], who found that Cipralex increases bone sclerostin and DKK-1 levels, indicating decreased bone formation. They also used micro-computed tomography of the lumbar bone to observe a significant decrease in bone mineral density and a change in bone microarchitecture.

The statistically significant drop in the percentage of newly generated collagen area in the Cipralex group may be a symptom of either increased collagen degradation or decreased collagen formation.

TNF-α and IL-1β are well-known proinflammatory cytokines linked to depression, with increased levels associated with disease severity and symptom persistence [59, 60]. SSRIs, including cipralex, appear to impact these cytokines, albeit the effects differ between research. Meta-analytic evidence suggests that antidepressant treatment in depressive disorders fails to substantially decrease TNF‑α but is correlated to declines in IL‑1β, supporting the selective anti-inflammatory effects observed in the current study, where escitalopram did not alter TNF‑α but lowered IL‑1β levels [60, 61].

This pattern is consistent with study findings which show considerable reductions in IL-1β and other pro-inflammatory markers following SSRI treatment, although changes in TNF-α are less consistent [62]. Some in vitro studies report increased pro-inflammatory cytokine production under specific conditions, highlighting variability depending on treatment context and cellular environment [39]. In harmony, these results imply that cipralex may have selective immunomodulatory effects, especially on IL-1β, which may help explain its therapeutic effectiveness in treating depression, but TNF-α responses are still more variable and subject to individual and methodological influences.

The study has certain limitations because it is an animal experiment with implications for human work conditions, in addition, the current study only analyzed male rats. This study has various limitations that must be addressed when evaluating the results. Inflammatory cytokines were evaluated at a single endpoint and exclusively in serum samples. While this approach sheds light on the systemic inflammatory response associated with SSRI (Cipralex) delivery, it does not allow for the assessment of temporal changes in cytokine levels during treatment. Furthermore, the lack of cytokine measures in the alveolar bone and salivary gland tissues hinders the ability to differentiate between local paracrine/autocrine effects and systemic endocrine signaling. Serum cytokine levels may not fully reflect the local inflammatory conditions found in bone or glandular tissues, where cytokines play an important role in tissue remodeling and degeneration. Therefore, it is not possible to definitively establish a clear mechanistic relationship between the histopathological changes found in the mandibular bone and salivary glands and the observed systemic cytokine modifications. To better understand the local and systemic inflammatory pathways causing SSRI-induced oral and skeletal tissue modifications, future research utilizing tissue-specific cytokine analysis and multiple time-point evaluations is necessary.

## Conclusion

This study shows that administering cipralex causes structural changes in major SGs, such as impaired ductal and acinar architecture, and is linked to a lower percentage of newly formed collagen area. Furthermore, biochemical study demonstrated a selective drop in IL-1β levels without substantial changes in TNF-α, and histological analysis of alveolar bone revealed evidence of bone resorption. These results imply that long-term escitalopram use may be involved in selective control of pro-inflammatory cytokines and localized tissue alternation. These findings emphasize the necessity of monitoring dental health and salivary gland function in individuals undergoing long-term Cipralex therapy. Future research is necessary to investigate the processes underlying these tissue-specific effects and determine whether preventive measures may minimize possible negative consequences.

## Data Availability

The datasets used and/or analyzed during the current study are available from the corresponding author on reasonable request.

## Abbreviations

ADs: Antidepressants
CSF: Cerebrospinal fluid
CT: Connective tissue
ED: Excretory duct
EDTA: Ethylene diamine tetra-acetic acid
ELISA: Enzyme-Linked Immunosorbent Assay
GCTs: Granular convoluted tubules
H&E: Hematoxylin and eosin-stained sections
ID: Intercalated duct
IL-1β: Interleukin 1 beta
MT: Masson’s trichrome stain
OD: Optical density
RBCs: Red blood cells
ROS: reactive oxygen species
SSRI: Selective serotonin reuptake inhibitors
SD: Striated duct
SGs: Salivary glands
SLG: Sublingual gland
SMG: Submandibular gland
TCAs: Tricyclic antidepressants
TNF-α: Tumor necrosis factor alpha
US: United states
